# Application of a modified drop method for high-resolution pachytene chromosome spreads in two *Phalaenopsis* species

**DOI:** 10.1186/s13039-016-0254-8

**Published:** 2016-06-06

**Authors:** Yi-Tzu Kuo, Hui-Lan Hsu, Chih-Hsin Yeh, Song-Bin Chang

**Affiliations:** Department of Life Sciences, National Cheng Kung University, No.1, University Road, Tainan City, 701 Taiwan; Taoyuan District Agricultural Research and Extension Station, Council of Agriculture, Executive Yuan, 139, Sec. 2, Dongfu Rd., Houzhuang, Sinwu Dist., Taoyuan City, 32745 Taiwan

**Keywords:** *Phalaenopsis*, Orchid, Pachytene chromosome, Fluorescence in situ hybridization (FISH), FISH mapping, Drop method

## Abstract

**Background:**

Preparation of good chromosome spreads without cytoplasmic contamination is the crucial step in cytogenetic mapping. To date, cytogenetic research in the Orchidaceae family has been carried out solely on mitotic metaphase chromosomes. Well-spread meiotic pachytene chromosomes can provide higher resolution and fine detail for analysis of chromosomal structure and are also beneficial for chromosomal FISH (fluorescence in situ hybridization) mapping. However, an adequate method for the preparation of meiotic pachytene chromosomes in orchid species has not yet been reported.

**Results:**

Two Taiwanese native *Phalaenopsis* species were selected to test the modified drop method for preparation of meiotic pachytene chromosomes from pollinia. In this modified method, pollinia were ground and treated with an enzyme mixture to completely remove cell walls. Protoplasts were resuspended in ethanol/glacial acetic acid and dropped onto a wet inclined slide of 30° from a height of 0.5 m. The sample was then flowed down the inclined plane to spread the chromosomes. Hundreds of pachytene chromosomes with little to no cytoplasmic contamination were well spread on each slide. We also showed that the resolution of 45S rDNA-containing chromosomes at the pachytene stage was up to 20 times higher than that at metaphase. Slides prepared following this modified drop method were amenable to FISH mapping of both 45S and 5S rDNA on pachytene chromosomes and, after FISH, the chromosomal structure remained intact for further analysis.

**Conclusion:**

This modified drop method is suitable for pachytene spreads from pollinia of *Phalaenopsis* orchids. The large number and high-resolution pachytene spreads, with little or no cytoplasmic contamination, prepared by the modified drop method could be used for FISH mapping of DNA fragments to accelerate the integration of cytogenetic and molecular research in *Phalaenopsis* orchids.

## Background

Preparation of chromosome spreads is the foundation of cytogenetic research. Especially for the chromosomal mapping techniques, FISH (fluorescence in situ hybridization) or GISH (genomic in situ hybridization), well spread and intact chromosomes without interference from cytoplasm are critical to success. Additionally, the resolution of cytogenetic mapping depends on the degree of chromosome condensation. Compared with mitotic metaphase chromosomes, the meiotic counterparts at the pachytene stage are much less condensed and reveal a clear distribution pattern of heterochromatic and euchromatic regions. Hence, using pachytene chromosomes greatly enhances the resolution of chromosome mapping and also benefits chromosome identification based solely on chromosomal morphology.

High-resolution FISH mapping on pachytene chromosomes is a powerful technique to help integrate physical and genetic maps and also to evaluate genome assembly quality. This strategy has been successfully demonstrated in plant species, for example tomato [1, 2], potato [3], cucumber [4], *Rosa* [5] and *Amborella* [6]. With the increasing use of sequencing, sequence datasets, such as transcriptomes [7, 8], BAC end sequences [9], and genomic shotgun sequences [10] have been generated for many orchid species, especially for *Phalaenopsis* orchids. However, molecular genetic information has not yet been integrated with cytogenetic information for these species.

Current chromosomal research in orchids, such as FISH mapping of repetitive sequences and rDNA in *Dendrobium* [11], *Paphiopedilum* [12] and *Cymbidium* [13] and also GISH analysis in *Phalaenopsis* [14] and *Paphiopedilum* [15], has mainly been carried out on mitotic metaphase chromosomes. Therefore, advanced research, like analysis of chromosomal structure and integration of orchid molecular and cytogenetic research through FISH mapping, might be restricted because of the poor resolution of metaphase chromosomes, especially for the orchid species with small chromosomes.

Certain natural characteristics of some orchid species, such as short flowering period, small number of flowers and small pollinia, limit material supply and further restrict chromosomal research on meiotic chromosomes. Further, the specialized pollinium, one of the main features of the Orchidaceae family, is composed of a large number of compactly gathered pollen mother cells (PMCs). The pollen masses enhance the difficulty in digesting the cell wall of PMCs evenly and preparing meiotic chromosome spreads.

The most common methods for preparing meiotic spreads are the squashing method [16, 17], the spreading method [18, 19] and the drop method [16, 20–23]. The squashing method was developed mainly for chromosome counting in plant species. The spreading method was reported to be suitable for plants with small chromosomes and has been applied in preparing mitotic chromosomes of rice, maize and tomato [18, 19, 24, 25]. The drop method has several advantages over the squashing method, despite the large quantity of materials required. Several slides with similar quality can be prepared from the same batch of cell suspension and chromosomes are more likely to spread apart from the cytoplasm. Without the interference of cytoplasm, the success of FISH or GISH mapping is improved. Moreover, both mitotic metaphase and meiotic pachytene chromosomes are often less distorted when prepared by the drop method compared with those prepared by the squashing method.

The spreading methods reported in the previous studies [19, 26, 27] were successfully applied to prepare chromosome spreads for FISH mapping in tomato and *Erycina pusilla* in our laboratory. The pachytene chromosome spreads of *Phalaenopsis* species prepared following the reported protocols usually showed weak staining with DAPI or even lost after FISH. In addition, the conventional squashing method is incapable for preparing well-spread pachytene chromosomes from compactly gathered pollinia. Moreover, most previously reported drop methods were most solely applied in preparing mitotic metaphase chromosomes [21, 22], instead of less condensed and longer pachytene chromosomes. The recently developed ‘SteamDrop’ method was reported to be applicable in preparing both metaphase and pachytene chromosomes in wide range of species, however, technical expertise is demanded to get high-quality chromosome spreads [28].

In this study, we selected two Taiwanese native *Phalaenopsis* species as materials to develop and present the easily-mastered modified drop method for preparing high-resolution pachytene spreads. Additionally, the differences in chromosomal condensation level between chromosomes at the metaphase and pachytene stages in *Phalaenopsis* species were compared. Furthermore, 45S rDNA and 5S rDNA were mapped to demonstrate the applicability of the developed chromosome spreads in FISH mapping and analysis of chromosomal structure.

## Methods

### Plant materials

Two native *Phalaenopsis* species in Taiwan, *P. aphrodite* subsp*. formosana* (2n = 2× = 38) and *P. equestris* (2n = 2× = 38)*,* were selected as plant materials for preparation of chromosome spreads. Plants of both species were planted in a greenhouse and grown to flowering. Flower buds with size ranging from 8.50–9.20 mm and 4.50–5.20 mm of *P. aphrodite* subsp*. formosana* and *P. equestris*, respectively, were collected to obtain developing pollinia undergoing meiosis with chromosomes at the pachytene stage. Root tips of *P. aphrodite* subsp*. formosana* were collected and treated with 2 mM 8-Hydroxyquinoline at 15 °C for 3 h. Pollinia and root tips were then fixed in freshly prepared Carnoy’s fixative (glacial acetic acid: ethanol, 1:3 (v/v)) overnight at room temperature. After washing in distilled water for 5 min twice, they were stored in 70 % ethanol at −20 °C until use. A small piece of fixed pollinia was stained with 1 % acetocarmine solution and was squashed immediately for examination of the chromosomal stage of PMCs.

### The modified drop method for preparation of meiotic pachytene spreads

Pollinia with chromosomes at the pachytene stage were first rinsed in distilled water for 5 min twice and transferred to 10 mM citrate buffer (4 mM citric acid and 6 mM tri-sodium citrate, pH 4.5). The pollinia were dipped in 10 mM citrate buffer, separated from anther cap and ground into small pieces using a tissue grinder in a microcentrifuge tube. The dispersed small pieces of pollinia were digested in enzyme mixture (10 mM citrate buffer containing 1 % (w/v) pectinase solution (Sigma), 1 % (w/v) pectolyase Y23 (Seishin Pharmaceutical) and 1 % (w/v) cellulose Onozuka RS (Yakult Pharmaceutical)) at 37 °C for 30–45 min (*P. aphrodite* subsp*. formosana*) or 60–75 min (*P. equestris*). The sample was washed twice by adding distilled water to the digestion mixture, centrifuging for 12 min at 7,000 rpm, and discarding the supernatant. Distilled water was then substituted with freshly prepared Carnoy’s fixative and the same centrifugation condition (7,000 rpm, 12 min) and the substitution steps were repeated three times. The pellet was finally resuspended with 200 μl Carnoy’s fixative. The suspension was dropped onto a wet, acid-treated and inclined slide of 30° from a height of 0.5 m using a sigmacote-treated glass dropper. The sample was then allowed to flow down the inclined plane to spread the chromosomes. After air drying, these slides were further fixed in Carnoy’s fixative and then dehydrated in 95 % ethanol for 3 min. Meiotic spreads with little to no cytoplasm and good pachytene chromosome spreading were selected and stored at 4 °C for later use.

### DNA probe preparation

DNA fragments of 5S rDNA were amplified by PCR using forward primer 5S-F (5’-GAT CCC ATC AGA ACT TC-3’) and reverse primer 5S-R (5’-GGT GCT TTA GTG CTG GTA T-3’). PCR was performed using 50 ng genomic DNA, 100 μM of dNTP, 0.2 μM each of forward and reverse primer, 1× PCR buffer and 2.5 U FastStart Taq DNA polymerase (Roche) in a total volume of 50 μl. The PCR cycling conditions followed the steps: 95 °C for 5 min; 30 cycles of 95 °C 30 s, 55 °C for 2 min and 72 °C for 30 s; final elongation at 72 °C for 5 min. The DNA clone pTa71 containing 45S rDNA of *Triticum aestivum* cloned into vector pAC184 was used to detect the position of 45S rRNA gene on pachytene chromosomes. Plasmid DNA was extracted using Plasmid Mini Prep Purification Kit (GMbiolab). The PCR product of 5S rDNA and plasmid DNA were labeled with either biotin-dUTP or digoxigenin-dUTP by standard nick translation following the manufacturer’s protocol (Roche).

### Fluorescence in situ hybridization (FISH)

FISH was carried out as previously described with some modifications [19]. The selected slides were incubated at 37 °C overnight or 65 °C for 30 min to air-dry the chromosome spreads before FISH. Pre-hybridization treatment included 5 μg/ml pepsin for 20 min and freshly prepared formaldehyde buffer (1× PBS, 50 mM MgCl_2_ and 1 % formaldehyde) for 10 min. Slides were then dehydrated through an ethanol series (70, 90 and 100 %). The hybridization mixture (10 % dextran sulfate sodium, 50 % formamide, 2× SSC, 0.25 % SDS and 100–200 ng probe DNA) was boiled for 10 min, placed on ice for 5–10 min and added onto the slides. The slides were treated at 80 °C for 2.5 min on a hot plate and incubated in a humid chamber at 37 °C for 12–16 h. Digoxigenin-labeled probes were detected with sheep fluorescein isothiocyanate (FITC)-conjugated anti-digoxigenin antibody (Roche) and amplified with anti-sheep-FITC (VECTOR Laboratories), whereas biotin-labeled probes were detected and amplified with Avidin Texas-red (VECTOR Laboratories) and Biotinylated Anti-avidin D (VECTOR Laboratories), respectively. Finally, slides were dehydrated through an ethanol series and dried at 37 °C for 20 min. Chromosomes were counterstained with 1.5 μg/ml 4’, 6-diamidino-2-phenylindole (DAPI) in mounting medium (VECTOR Laboratories).

### Image capturing and cytological analysis

Images were captured using a Nikon DS Ri1 CCD camera attached to a Nikon ECLIPSE 80i microscope. Image adjustment and chromosomal straightening were carried out using ImageJ software. The length of the chromosome and the distance from the signal to the end of the chromosome were measured using NIS-Elements microscope imaging software (Nikon). The relative distance to the signal was calculated by the formula: Distance from the end of the short arm to the signal × 100 %/length of the pachytene chromosome.

## Results

### Collection of flower buds and examination of chromosome stage in PMCs

**Fig. 1:**
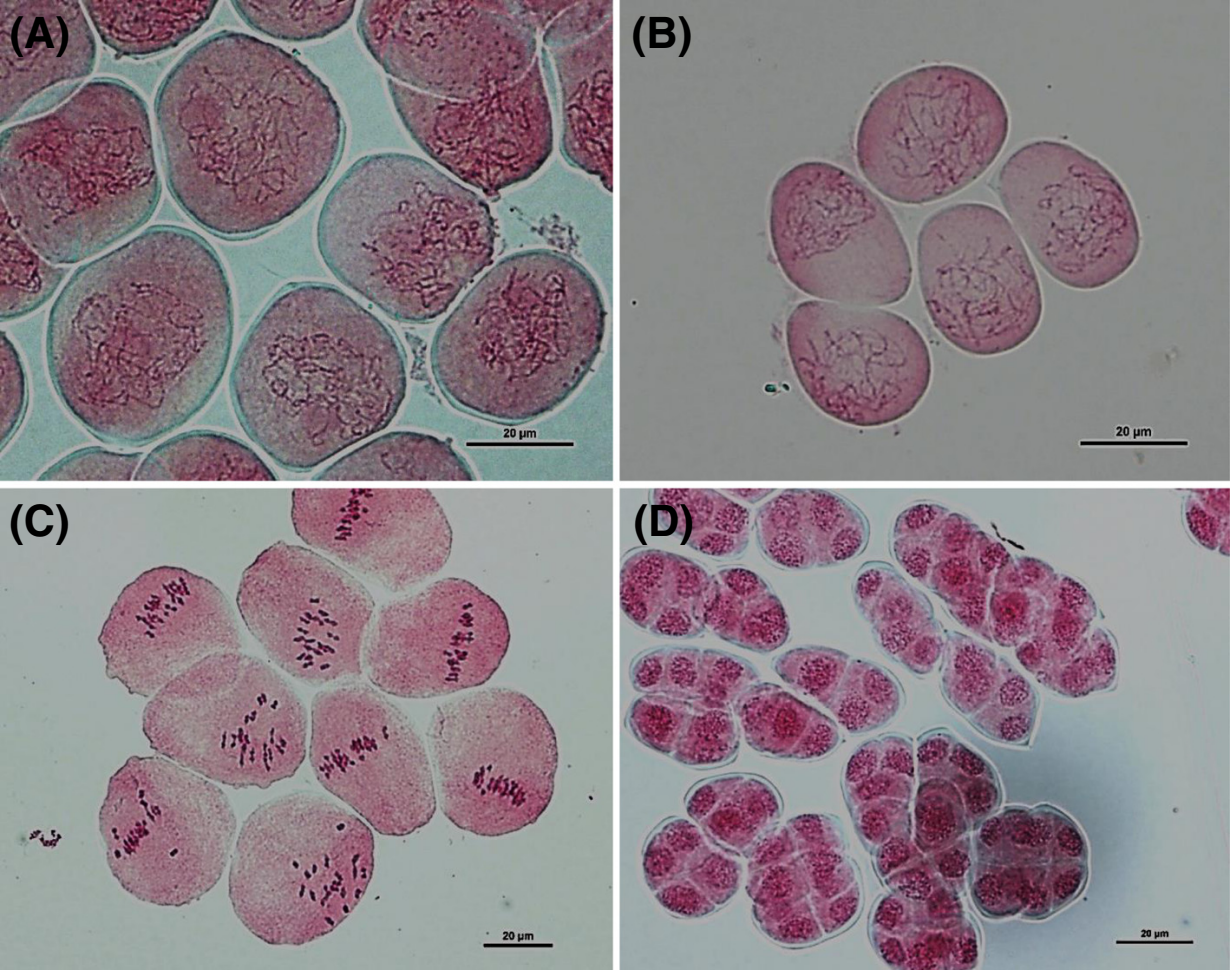
Examination of meiotic chromosome stage in pollen mother cells (PMCs). Pachytene stage in *P. aphrodite* subsp*. formosana* (**a**) and in *P. equestris* (**b**). Chromosomes at prometaphase I, metaphase I (**c**) and tetrad stages (**d**) of PMCs from *P. aphrodite* subsp*. formosana.* PMCs were stained with 1 % acetocarmine, scale bar = 20 μm

Flower buds, with sizes ranging from 8.50–9.20 mm for *P. aphrodite* subsp*. formosana* and 4.50–5.20 mm for *P. equestris,* were examined, with most PMCs having mostly meiotic chromosomes at the pachytene stage (Fig. 1a–b). However, PMCs with chromosomes at the zygotene or diakinesis stages were also observed in these flower buds. Most of the flower buds of *P. aphrodite* subsp*. formosana* with size over 9.50 mm had PMCs at metaphase I or even at the tetrad stage (Fig. 1c–d). For *Phalaenopsis* species, pollinia contain two halves of the pollinium attached to an anther cap. Sometimes asynchronous cell division of PMCs between the two halves of the pollinium was observed.

### Preparation of meiotic chromosome spreads using the modified drop method

**Fig. 2:**
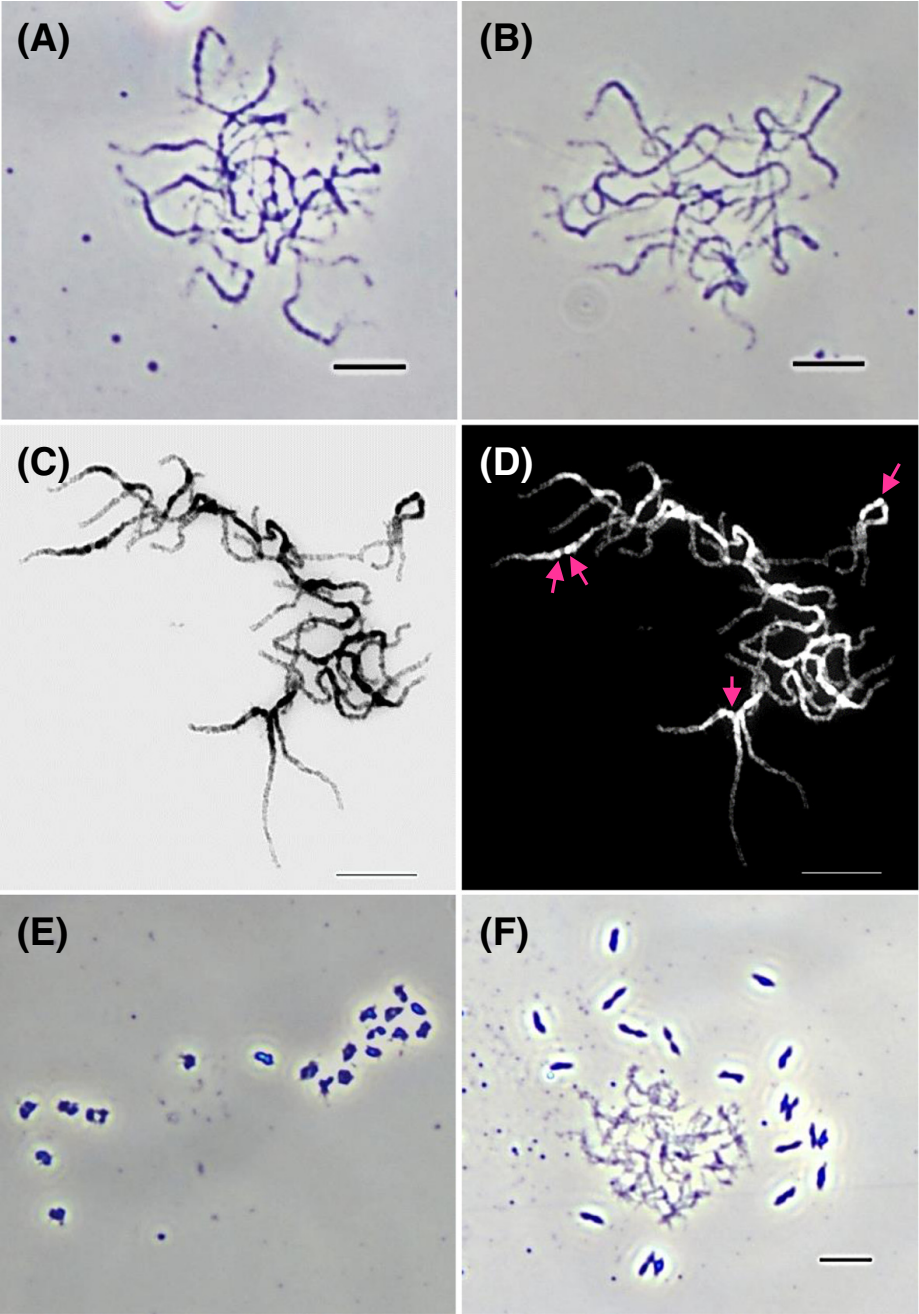
*Phalaenopsis* meiotic spreads prepared by the modified drop method. Pachytene spread of *P. equestris* (**a**) and *P. aphrodite* subsp*. formosana* (**b**). **c**-**d** The DAPI-staining pachytene chromosomes of *P. aphrodite* subsp*. formosana* were converted to *black* and *white* to enhance the visualization of chromosomal structure. The *pink arrows* in (**d**) indicate chromomeres on pachytene chromosomes. Chromosomes of *P. equestris* (**e**) and *P. aphrodite* subsp*. formosana* (**f**) at meiotic metaphase I. Scale bar = 10 μm

Owing to the loss of some PMCs during chromosome preparation, particularly in protoplast preparation, pollinia from one *P. aphrodite* subsp*. formosana* flower bud or 2–3 pollinia of *P. equestris* were used to prepare 15–25 slides with similar quality. A tissue grinder was used to crush masses of PMCs separated from the anther cap and also to disperse pollinia into uniformly small pieces in a microcentrifuge tube. To eliminate the cell wall of PMCs, the same enzyme mixture was applied in the two species. The optimal enzyme treatment incubation was found to be 30–45 min and 60–75 min for *P. aphrodite* subsp*. formosana* and *P. equestris*, respectively. After such treatment, over 80 % (visual observation) of meiotic spreads were free from interference of wall fragments and cytoplasm. Slight interference from the cytoplasm might have no effect on further chromosome staining but pre-treatment with pepsin might be needed before FISH hybridization to facilitate probe penetration. The cell suspensions of the two *Phalaenopsis* species were dropped onto wet slides from a height of 0.5 m and most of the pachytene chromosomes were structurally intact and also well spread (Fig. 2a–b). Approximately 100–250 meiotic pachytene spreads were obtained on each slide following this modified drop method. Converting images of DAPI-stained pachytene chromosomes into black-and-white with a white background enhanced the visual contrast between heterochromatic and euchromatic regions (Fig. 2c). However, brightly stained chromomeres on some pachytene chromosomes of *P. aphrodite* subsp*. formosana* were easily observed in an image with a black background (Fig. 2d).

Meiotic spreads at stages other than pachytene, like metaphase I, can also be prepared using this method. Nineteen non-overlapping tetrads of *P. equestris* (Fig. 2e) and *P. aphrodite subsp. formosana* (Fig. 2f) at metaphase I are shown. Adjustment of the dropping height might avoid overlap of the highly condensed chromosomes.

### FISH mapping of 45S rDNA and comparison of chromosome condensation at metaphase and pachytene stages

**Fig. 3:**
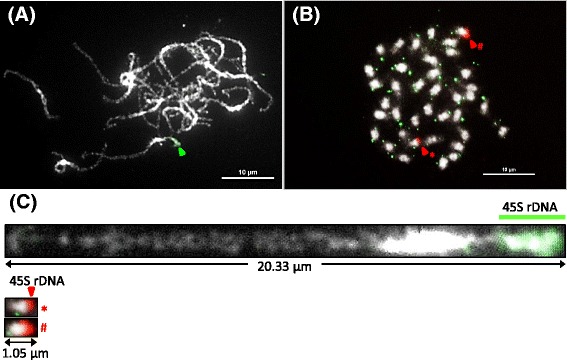
FISH mapping of 45S rDNA (*arrowheads*) on chromosomes at pachytene stage (**a**) and metaphase (**b**) in *P. aphrodite* subsp*. formosana*. Telomere DNA in green (**b**) was also mapped on metaphase chromosomes. **c** The pachytene and metaphase chromosomes with 45S rDNA signals were straightened using ImageJ software. The *asterisk* and pound sign indicate the corresponding chromosomes in (**b**). Chromosomes were stained with DAPI and images were converted to *black* and *white*. Scale bar = 10 μm

The basic chromosome number of *Phalaenopsis* species is up to x = 19. Therefore, it is relatively hard to trace and identify every chromosome in a single cell, especially the more tangled pachytene chromosomes, and to measure the total complement length. To compare the levels of chromosome condensation at different stages, 45S rDNA was mapped on both metaphase and pachytene chromosomes of *P. aphrodite* subsp*. formosana* using FISH, and the length of this 45S rDNA-containing chromosome was measured. First, the applicability of chromosome spreads prepared by this modified drop method to FISH mapping was proved. Second, a hybridization signal of 45S rDNA was detected on one end of a meiotic pachytene chromosome (Fig. 3a) and a pair of signals was observed on two mitotic metaphase chromosomes in *P. aphrodite* subsp*. formosana* (Fig. 3b). 45S rDNA is a highly repetitive locus in the plant genome. A strong signal for 45S rDNA was observed and can be detected on almost every chromosome spread prepared by this method. Fourteen 45S rDNA-containing pachytene chromosomes were straightened; their length ranged from 19.76 μm to 25.84 μm (average 22.06 μm), while the length of this chromosome at metaphase was only about 1.05 μm (Fig. 3c). This result indicated that chromosomes at the meiotic pachytene stage provide over 20 times the resolution of those at mitotic metaphase in *P. aphrodite* subsp*. formosana*. We were also able to observe that most of the 45S rDNA-containing chromosome was euchromatin except for the bright DAPI-staining pericentromeric heterochromatin and the NOR (nucleolus organizer region) on the end of the short arm (Fig. 3c). This demonstrates that the distribution pattern of heterochromatin and euchromatin on pachytene spreads prepared by this method can be clearly distinguished by DAPI staining.

### Precise location of 5S rDNA on pachytene chromosomes

**Fig. 4:**
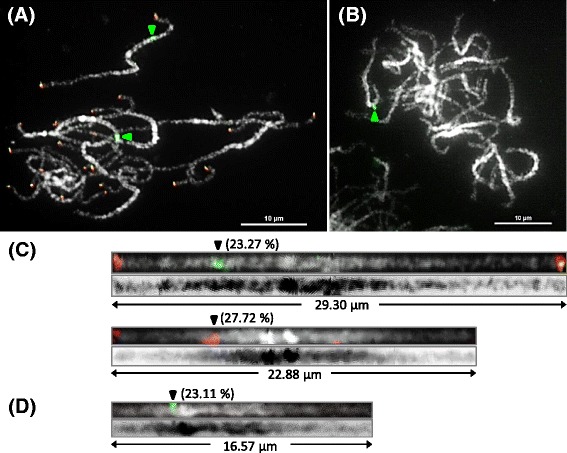
FISH mapping of 5S rDNA on pachytene chromosomes. **a** Two distinct signals of 5S rDNA (*green arrowheads*) were observed in *P. aphrodite* subsp*. formosana* and the red signals represent telomeres. **b** Only one signal of 5S rDNA (*green arrowhead*) was detected in *P. equestris.* The 5S rDNA-located chromosomes of *P. aphrodite* subsp*. formosana* (**c**) and *P. equestris* (**d**) were straightened using ImageJ and arrowheads indicated the signals of 5S rDNA. The relative distance of a signal on a pachytene chromosome was shown as a percentage. The images of DAPI-stained chromosomes were converted to *black* and *white*. Scale bar = 10 μm

The 5S rDNA of both *P. aphrodite* subsp*. formosana* and *P. equestris* was amplified and mapped on pachytene chromosomes by FISH to show its precise location. Obvious signals of 5S rDNA were detected on two pachytene chromosomes of *P. aphrodite* subsp*. formosana* (Fig. 4a) and only one signal presented in *P. equestris* pachytene chromosomes (Fig. 4b). Each of these 5S rDNA-containing pachytene chromosomes were traced and straightened. The signals of 5S rDNA were all mapped on the short arm of these chromosomes. The relative distances of the 5S rDNA signals on the two pachytene chromosomes of *P. aphrodite* subsp*. formosana* were 23.27 and 27.72 % (Fig. 4c) and the relative distance of the signal in *P. equestris* was 23.11 % (Fig. 4d). Noticeably, the two 5S rDNA-containing chromosomes of *P. aphrodite* subsp*. formosana* can be easily discriminated owing to the presence of two obvious chromomeres on one of these two chromosomes (Fig. 4c). In summary, the pachytene spreads prepared by the modified drop method can be applied in FISH mapping, with chromosomal structure remaining intact after FISH for further analysis.

## Discussion

### Advantages of the modified drop method

The drop method was first developed for preparing chromosome spreads of human cells [29] and for protoplast isolation in plant species [30]. Since then, several methods have been published to improve the techniques of meiotic chromosome preparation for plant species [19, 28, 31, 32], but none of these methods has been reported suitable for orchid species with specialized pollinia. In this study, we developed the first easily mastered drop method for preparation of high-quality meiotic pachytene chromosomes in *Phalaenopsis* species. The protocol of drop preparation of plant chromosomes [33] combined with the other two reported methods [34, 35] were taken as the original protocols to develop the modified drop method. In this method, tissue grinders are used to crush specialized *Phalaenopsis* pollinia into scattered PMCs for eliminating cell walls completely. Repeat times of centrifugation/resuspension and speed of centrifugation are optimized to decrease the amount of dirt in suspension and avoid excessive sample loss. The adequate dropping height is adjusted to bombard the cell suspension onto the wet slides for *Phalaenopsis* species. The samples then flow down freely along the inclined plane to spread chromosomes, instead of blowing or spraying bursting medium, which is relatively more technical expertise demanded. Therefore, the advantages of this method over the previous methods include: (1) the cell walls of tightly compacted PMCs can be uniformly eliminated; (2) no technical expertise is needed for dropping protoplasts onto inclined slides using a dropper, or spreading chromosomes; (3) most chromosome spreads are free from contamination by wall fragments and cytoplasm; (4) several slides with similar quality can be prepared in a single experiment; (5) up to 100 meiotic pachytene spreads can be obtained on each slide; (6) the chromosomal structure remains intact even after FISH when samples are dropped from the optimized dropping height. However, different numbers of pollinia may be required from different species to obtain sufficient PMCs for spread preparation following this protocol. In *Phalaenopsis* species like *P. equestris*, two to three pollinia were required to prepare slides with a similar spread number to *P. aphrodite* subsp*. formosana* using pollinia from only one flower bud. Additionally, asynchronous cell divisions of PMCs in the two halves of the pollinium were sometimes found. Hence, examining the chromosomal stage of PMCs in both halves of the pollinium, before preparation of meiotic chromosome spreads, is recommended to avoid getting chromosome spreads at different division stages on the same slide.

### High resolution of meiotic pachytene chromosomes of *Phalaenopsis* species

The length differences of chromosome counterparts at pachytene and metaphase stages among different plant species are diverse. The chromosomes at the pachytene stage are much longer than those at metaphase, with a 7-fold length difference for rye, 10-fold for maize, 15-fold for tomato, over 20-fold for *Arabidopsis* and up to 40-fold for rice [36]. According to the length analysis of the 45S rDNA-containing chromosome, the length of the chromosomes at meiotic pachytene was over 20-fold longer than that at mitotic metaphase in *P. aphrodite* subsp*. formosana*. A pair of 5S rDNA signals was detected on the end of two mitotic metaphase chromosomes in *P. equestris* [37]. While mapping of 5S rDNA revealed one signal on the meiotic pachytene chromosomes, our result showing only one 5S rDNA locus in *P. equestris* genome was consistent with previous studies. However, the relative distance of 5S rDNA signal on the chromosome at the pachytene stage was 23.27 %, not on the end as is suggested by analysis of metaphase chromosomes. This demonstrates that the higher resolution of the meiotic pachytene chromosome makes it the stage of choice to detect the precise location of DNA fragments on chromosomes. Moreover, pachytene spreads of *P. aphrodite* subsp*. formosana* prepared by the modified drop method preserved obvious chromomere structures on certain chromosomes, including two chromomeres on one of the 5S rDNA-containing chromosomes. The chromomeres on pachytene chromosomes can be taken as unique markers for chromosome identification. Together these results suggest that preparation of *Phalaenopsis* pachytene chromosomes, with higher resolution and obvious structural characteristics, will benefit further cytogenetic research in *Phalaenopsis* species.

### Further applicable prospects of this modified drop method

The first draft genome sequence of the Orchidaceae family was determined in *P. equestris* [10], but the assembled scaffolds have not yet been mapped to chromosomes or linkage groups. Cytogenetic studies of *Phalaenopsis* species are relatively rare and karyotypes of the few *Phalaenopsis* species investigated were constructed based solely on metaphase chromosomes [38]. High-resolution pachytene chromosomes have not been prepared and investigated in *Phalaenopsis* cytogenetic research. High-quality pachytene spreads can be easily prepared using this modified drop method and well-dispersed pachytene chromosomes are amenable for establishing high-resolution karyotypes. The pachytene chromosome-based karyotype has been established in several species like cucumber [39], tomato [27], potato [3], papaya [40] and *Daucus* species [41], but not yet in *Phalaenopsis* species. Slides with large numbers of pachytene spreads combined with well-developed FISH mapping techniques are suitable for large-scale cytogenetic analysis to help verify the quality of genome assembly and integrate physical and genetic maps. This modified drop method could be further used to prepare meiotic chromosome spreads from pollinia of orchid species other than *Phalaenopsis* species, although the conditions of dropping height and enzyme treatment for different species, or preparing spreads of chromosomes at different stages must be optimized. Moreover, laser capture microdissection is an increasingly popular technique to isolate specific chromosomal regions or DNA fragments spread on membrane-coated slides [42]. The modified drop method could also be used to prepare chromosome spreads on membrane-coated slides, with less contamination and therefore less interference in downstream applications such as DNA amplification.

## Conclusions

Here we describe and demonstrate for the first time a modified drop method for the preparation of pachytene chromosome spreads from the specialized pollinia in *Phalaenopsis* orchids. The up to 20-fold higher resolution of chromosomes at the pachytene, compared with the metaphase, stage, provides a marked improvement in the information that can be acquired using FISH mapping, resulting in improved cytogenetic investigations in *Phalaenopsis* species. In addition to the high resolution, the chromosome spreads prepared by this method can reveal clear patterns of heterochromatic and euchromatic regions and obvious chromomere structures. The applicability of the prepared chromosome spreads was also demonstrated by FISH mapping of both 45S and 5S rDNA. The signals of these rDNA probes were easily detected and the chromosomal structure after FISH remained intact for further analysis. Moreover, 15–25 slides of similar quality can be prepared from the same batch of cell suspension and 100–250 pachytene spreads can be obtained on one slide using this method. Combining the high-quality pachytene chromosomes with well-established FISH mapping techniques will result in a powerful tool for large-scale cytogenetic research, and will further accelerate the integration of molecular and cytogenetic research in *Phalaenopsis* and other orchid species.

## Abbreviations

DAPI, 4’, 6-diamidino-2-phenylindole; FISH, fluorescence in situ hybridization; FITC, fluorescein isothiocyanate; GISH, genome in situ hybridization; NOR, nucleolus organizer region; PBS, phosphate buffered saline; PMC, pollen mother cell; rDNA, ribosomal DNA; SDS, sodium docecyl sulfate; SSC, saline sodium citrate
